# Amplitude of Low Frequency Fluctuation Abnormalities in Adolescents with Online Gaming Addiction

**DOI:** 10.1371/journal.pone.0078708

**Published:** 2013-11-04

**Authors:** Kai Yuan, Chenwang Jin, Ping Cheng, Xuejuan Yang, Tao Dong, Yanzhi Bi, Lihong Xing, Karen M. von Deneen, Dahua Yu, Junyu Liu, Jun Liang, Tingting Cheng, Wei Qin, Jie Tian

**Affiliations:** 1 School of Life Science and Technology, Xidian University, Xi’an, Peoples R China; 2 Department of Medical Imaging, the First Affiliated Hospital of Medical College, Xi’an, Jiaotong University, Xi’an, Shaanxi, China; 3 Information Processing Laboratory, School of Information Engineering, Inner Mongolia University of Science and Technology, Baotou, Inner Mongolia, China; 4 Baotou Heping Middle School, Baotou, Inner Mongolia, China; 5 Institute of Automation, Chinese Academy of Sciences, Beijing, China; Emory University, United States of America

## Abstract

The majority of previous neuroimaging studies have demonstrated both structural and task-related functional abnormalities in adolescents with online gaming addiction (OGA). However, few functional magnetic resonance imaging (fMRI) studies focused on the regional intensity of spontaneous fluctuations in blood oxygen level-dependent (BOLD) during the resting state and fewer studies investigated the relationship between the abnormal resting-state properties and the impaired cognitive control ability. In the present study, we employed the amplitude of low frequency fluctuation (ALFF) method to explore the local features of spontaneous brain activity in adolescents with OGA and healthy controls during resting-state. Eighteen adolescents with OGA and 18 age-, education- and gender-matched healthy volunteers participated in this study. Compared with healthy controls, adolescents with OGA showed a significant increase in ALFF values in the left medial orbitofrontal cortex (OFC), the left precuneus, the left supplementary motor area (SMA), the right parahippocampal gyrus (PHG) and the bilateral middle cingulate cortex (MCC). The abnormalities of these regions were also detected in previous addiction studies. More importantly, we found that ALFF values of the left medial OFC and left precuneus were positively correlated with the duration of OGA in adolescents with OGA. The ALFF values of the left medial OFC were also correlated with the color-word Stroop test performance. Our results suggested that the abnormal spontaneous neuronal activity of these regions may be implicated in the underlying pathophysiology of OGA.

## Introduction

Online gaming addiction (OGA) is defined as a maladaptive use of the Internet and the inability of an individual to control his/her use of the Internet, which has been classified as one type of impulse control disorder [1]–[3]. Data from the China Youth Internet Association (announcement on February 2, 2010) demonstrated that the incidence rate of OGA among Chinese urban youths is about 14%. As one of the common mental health problems amongst Chinese adolescents, OGA has been associated with the impairment of the individual’s psychological well-being, academic failure and reduced work performance [4], which is currently becoming a more and more serious health problem in adolescents around the world [5], [6]. While OGA is not yet officially codified within a psychopathological framework, numerous studies of OGA adolescents have revealed structural and functional abnormalities in the orbitofrontal cortex (OFC), supplementary motor area (SMA), cingulate cortex, parahippocampal gyrus (PHG), dorsolateral prefrontal cortex (DLPFC), precuneus, temporal gyrus, insula and the cerebellum [1], [2] Abnormalities in these regions have been associated with substance abuse by numerous addiction studies [7], and may be associated with dysfunctions in cognitive control, executive control, craving, reward sensitivity, goal-directed behavior and working memory in OGA adolescents [1].

Although OGA causes individual and social burden, there is currently no standardized treatment for OGA [8]. Clinics in China have implemented regimented timetables, strict discipline and electric shock treatment, and gained notoriety for these treatment approaches [4]. Developing effective methods for intervention and treatment of OGA will require establishing a clear understanding of the mechanisms underlying this condition. To date, most OGA studies have focused on detecting structural deficits and task-related functional impairments in people with OGA, which were helpful in evaluating neural mechanisms underlying OGA. However, few studies have evaluated the blood oxygen level–dependent (BOLD) signal change of the regional spontaneous activity of OGA during the resting state. As a noninvasive approach, resting state functional magnetic resonance imaging (fMRI) has been utilized to investigate spontaneous low frequency fluctuations (LFF) in BOLD signals, which avoids performance-related confounds and can reflect spontaneous neural activity in the brain [9], [10]. Furthermore, the resting state fMRI method has been extensively used to reveal the intrinsic typical and atypical functional architecture of the brain [10]. The abnormal neuronal activity during resting state may serve as an adequate marker to reflect the progress and impaired executive function of multiple brain diseases.

Recently, Liu et al. employed the regional homogeneity (ReHo) method and found that people with OGA showed a significant increase in ReHo values in the right cingulate gyrus, bilateral parahippocampus, left precuneus, and left superior frontal gyrus [11]. The ReHo method reflects the temporal homogeneity of regional LFF regardless of the intensities, and is based on the hypothesis that spatially neighboring voxels should have similar temporal patterns [12]. While the amplitude of LFF (ALFF) is thought to be associated with local neuronal activity, the basis of ALFF changes in OGA remains unclear [13]. Moreover, Liu et al. [11] did not investigate the relationship between abnormal resting-state properties and duration of OGA. To further investigate the resting state abnormalities in OGA adolescents, the ALFF method was employed in the present study and data on the duration of OGA were collected. Furthermore, researchers have detected impaired cognitive control ability in adolescents with OGA using a color-word Stroop task [14], [15]. Therefore, the behavioral assessment in the present study was the performance on color-word Stroop task. The connection of neuroimaging findings to well-defined behavioral indices that are known to be affected in OGA would be a further index of the importance of these findings to OGA.

## Materials and Methods

All research procedures were approved by the West China Hospital Subcommittee on Human Studies and were conducted in accordance with the Declaration of Helsinki. All participants and their guardians in our study gave written informed consent.

### Subjects

According to the modified Young Diagnostic Questionnaire (YDQ) for OGA criteria by Beard and Wolf [8], [16], twenty students with OGA were filtered out from 165 freshman and sophomore students. Eighteen adolescents with OGA (12 males, mean age  =  19.4±3.1 years, education 13.4±2.5 years) participated in our study by excluding two left-handed players. To investigate whether or not there were any linear changes in brain structure, the duration of the disease was estimated via a retrospective diagnosis. We asked the subjects to recall their life-style when they were initially addicted to their mainly online game, i.e. World of Warcraft (WOW). To guarantee that they were suffering from OGA, we retested them with the YDQ criteria modified by Beard and Wolf. The reliability of the self-reports from the OGA subjects was also confirmed by talking with their parents via telephone as well as roommates and classmates.

Eighteen age- and gender-matched healthy controls (12 males and 6 females, mean age  =  19.5±2.8 years, education 13.3±2.0 years) with no personal or family history of psychiatric disorders also participated in our study. According to previous OGA studies, we chose healthy controls who spent less than 2 hours per day on the Internet [4]. The healthy controls were also tested with the YDQ criteria modified by Beard and Wolf to ensure they were not suffering from OGA. All recruited participants screened were native right-handed Chinese and were assessed by a personal self-report and Edinburgh Handedness Questionnaire. Exclusion criteria for both groups were 1) existence of a neurological disorder evaluated by the Structured Clinical Interview for the Diagnostic and Statistical Manual of Mental Disorders, Fourth Edition (DSM-IV); 2) alcohol, nicotine or drug abuse via urine drug screening; 3) pregnancy or menstrual period in women; and 4) any physical illness such as a brain tumor, hepatitis, or epilepsy as assessed according to clinical evaluations and medical records. The Hamilton anxiety scale (HAMA) and the Beck depression inventory-II (BDI) were used to evaluate the emotional states of all participants during the preceding two weeks. More detailed demographic information is given in Table 1.

**Table 1:** Subject demographics for adolescents with online gaming addiction (OGA) and control groups.

Items	OGA	Control	*P* value
	N = 18	N = 18	
Age (years)	19.4±3.1	19.5±2.8	>0.05
Gender	12 males 6 females	12 males 6 females	>0.05
Education (years)	13.4±2.5	13.3±2.0	>0.05
Duration of internet addiction (months)	34.8±8.5	N/A	N/A
Hours of internet use (/day)	10.2±2.6	0.8±0.4	**
Days of internet use(/week)	6.3±0.5	1.6±0.8	**
Hamilton anxiety scale	12.4±10.4	6.5±2.9	>0.05
Beck depression inventory	11.4±6.8	4.3±2.5	**

* : *p*<0.05; **: *p*<0.005.

### Behavioral data collection

According to a previous study [17], the color-word Stroop task design was implemented by using E-prime 2.0 software (http://www.pstnet.com/eprime.cfm). This task employed a block design with three conditions, i.e. congruent, incongruent and rest. Three words, Red, Blue, and Green were displayed in three colors (red, blue and green) as the congruent and incongruent stimuli. During rest, a cross was displayed at the center of the screen, and subjects were required to fix their eyes on this cross without responding. All events were programmed into two runs with different sequences of congruent and incongruent blocks. Each participant was instructed to respond to the displayed color as fast as possible by pressing a button on a Serial Response Box™ with the right hand. Button presses by the index, middle, and ring finger corresponded to red, blue, and green respectively. Participants were tested individually in a quiet room when they were in a calm state of mind. After the initial practice, the behavioral data was collected two or three days before MRI scanning.

### MRI Data Acquisitions

All fMRI studies were performed on a 3-T GE scanner (EXCITE, GE Signa, Milwaukee, WI, USA) using a standard birdcage head coil as an eight-channel phase-array head coil in the Huaxi MR Research Center, Chengdu, China. The foam pads were used to diminish head motion and scanner noise. After conventional localizer scanning, the T1-weighted images were obtained with a spoiled gradient recall sequence (repetition time (TR)  =  1900 ms; echo time (TE)  =  2.26 ms; flip angle (FA)  =  9°; field of view (FOV)  =  256×256 mm^2^; data matrix  =  256×256; slices  =  176; voxel size  =  1×1×1 mm^3^). Then, resting-state functional images were acquired using an echo-planar-imaging sequence (TR  =  2000ms; TE  =  30ms; FA  =  90°; FOV  =  240×240 mm^2^; data matrix  =  64×64) with 32 axial slices (slice thickness  =  5 mm and no slice gap, total volumes = 180) in one run of six minutes. The subjects were instructed to close their eyes, keep still and to not think about anything systematically during the scanning. At the end of the data acquisition, all subjects confirmed that they remained awake during the whole scanning period.

### Data preprocessing and ALFF calculation

All of the functional image processing was performed with Statistical Parametric Mapping (SPM5, http://www.fil.ion.ucl.ac.uk/spm) software and Data Processing Assistant for Resting-State fMRI (DPARSF) software [18]. For each participant, the first ten time points were discarded to avoid transient signal changes before magnetization reached steady-state and to allow subjects to get used to the fMRI scanning environment. The remaining 170 brain volumes were corrected for slice-timing and realigned for head movement correction. No subjects had head movement exceeding 1 mm of movement or 1° rotation in any direction. Then, all of the realigned images were spatially normalized into the Montreal Neurological Institute (MNI) EPI template, resampled to 3 mm isotropic voxels and then spatially smoothed (full-width at half-maximum  =  8 mm). After that, by calling functions in the Resting-State fMRI Data Analysis Toolkit (REST, http://rest.restfmri.net), linear-trend removal and band-pass filtering (0.01–0.08 Hz) for reducing the effects of low frequency drift and high frequency physiological noise [18] were performed on the time series.

After preprocessing, the ALFF calculation was performed using DPARSF by calling functions in REST as in previous studies [19]. First, for obtaining the power spectrum, the filtered time series was transformed to the frequency domain using a fast Fourier transform (FFT). Then the square root of the power spectrum was obtained for each frequency data point to yield amplitude as a function of frequency. These values, averaged across 0.01–0.08 Hz at each voxel, were used as the ALFF values. Consequently, this averaged square root was used as the ALFF value. The ALFF of each voxel was divided by the global mean ALFF value within the whole-brain mask for each subject, resulting in a standardized ALFF of each voxel which had a value of about 1.

### Statistical analysis

To assess the differences between the OGA group and control group in age, gender, disease duration, and years of education, two-sample *t*-tests were performed using SPSS 13.0 and a *p*>0.05 was deemed insignificant. To explore which areas had ALFF values differing from the value of 1, a one-sample *t*-test (*p*<0.05, family-wise error (FWE) corrected) using SPM5 was performed within each group. Then, a two-sample *t*-test was performed to elucidate ALFF differences between the two groups after controlling for age and gender. Correction for multiple comparisons was performed using Monte Carlo simulations. A corrected threshold of *p*<0.05 was derived from a combined threshold of *p*<0.005 for each voxel and a minimum cluster size of 351 mm^3^ (AlphaSim program in AFNI software, http:// afni.nimh.nih.gov/). For the brain regions in which OGA patients showed abnormal ALFF properties, the ALFF values of each region were extracted, averaged and regressed against the pathological indicators reflected by the duration of the disease and the color-word Stroop task performances.

## Results

Our results demonstrated that the rate of OGA was about 12.1% in our small sample investigation. According to their self-report of Internet use, the OGA subjects spent 10.2±2.6 hours per day and 6.3±0.5 days per week on online gaming. Adolescents with OGA spent more hours per day and more days per week on the Internet than the controls (*p*<0.005) (Table 1).

### Behavioral data results

Both groups showed a significant Stroop effect, where the reaction time was longer during the incongruent than the congruent condition (OGA: 677.3±75.4 ms vs 581.2±71.6 ms and controls: 638.3±65.9 ms vs 549.0±50.6 ms; *p*<0.005). The OGA group committed more errors than the control group during the incongruent condition (8.56±4.77 vs 4.56±2.93; *p*<0.05), although the response delay measured by reaction time (RT) during the incongruent condition minus congruent conditions was not significantly different between these two groups (98.2±40.37 ms vs 91.92±45.87 ms; *p* > 0.05).

### Imaging data results

**Figure 1:**
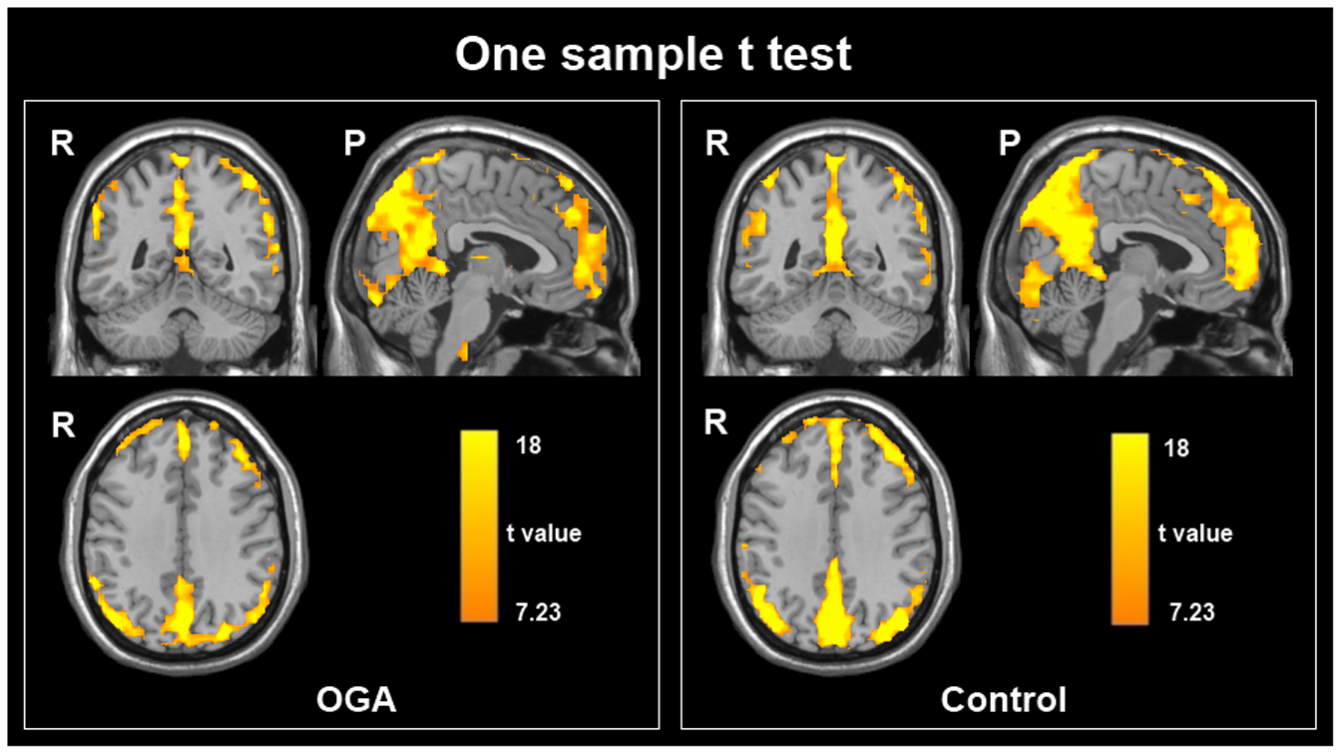
One sample *t*-test results. Within-group ALFF maps within the OGA and healthy control groups (*p*<0.05, FWE corrected, R, right; P, posterior).

**Figure 2:**
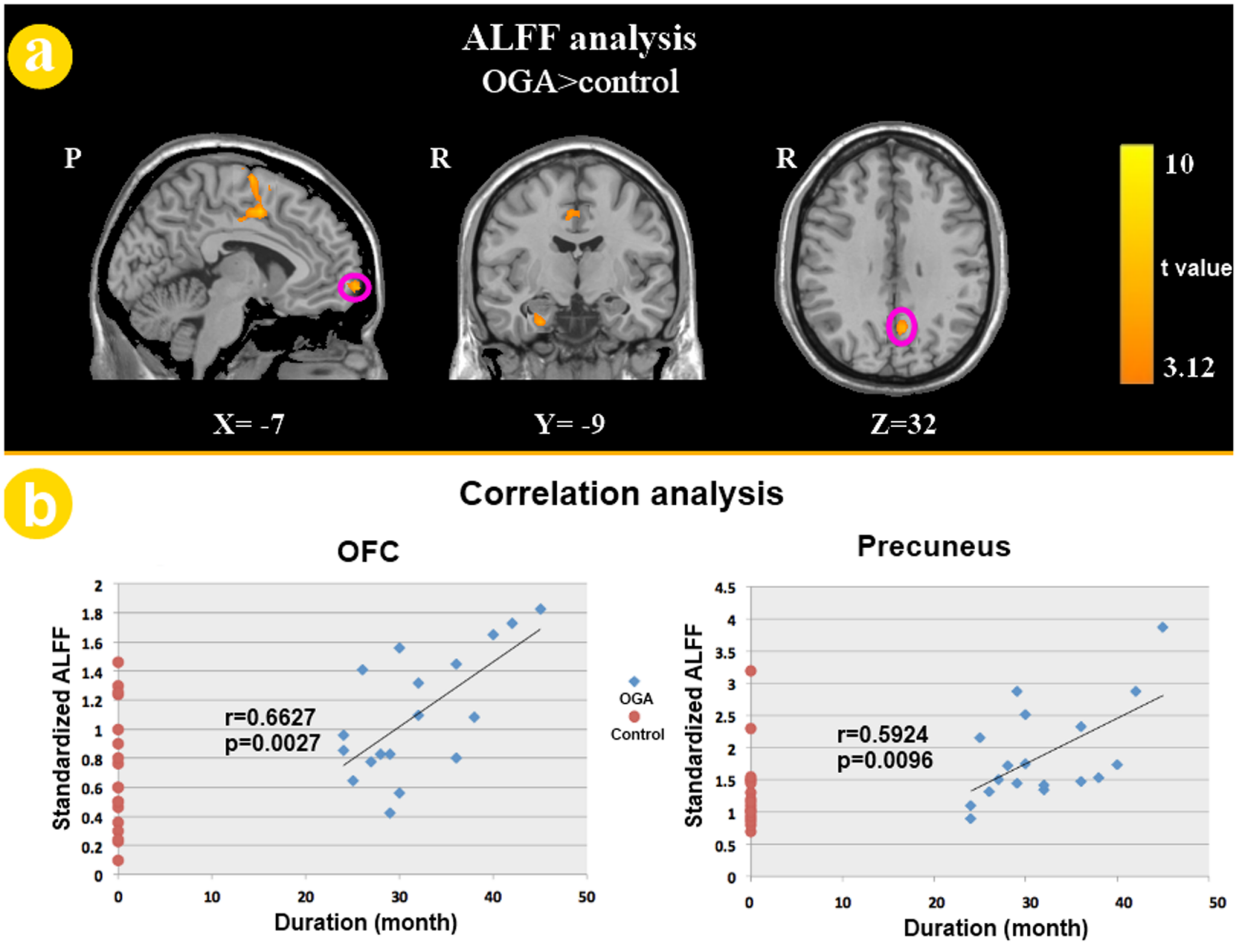
Two sample *t*-test analysis. (a) ALFF differences between OGA and healthy control groups (OGA>Controls, *p*<0.05, corrected). Warm colors indicate ALFF increases in patients with OGA. T-score bars are shown on the right. (b) The correlation analysis results between the standardized ALFF values of the left medial OFC, left precuneus and duration of the OGA. Abbreviation: medial orbitofrontal cortex (mOFC).

**Figure 3:**
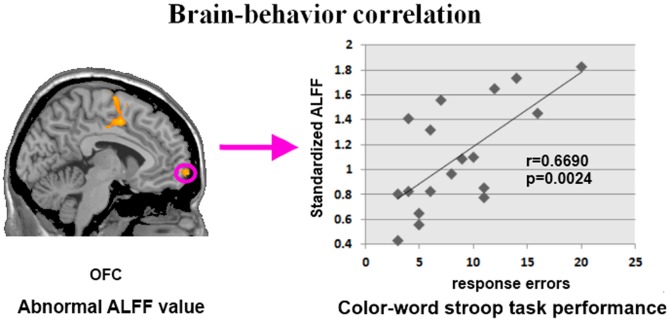
Brain-behavior relationship analysis. The ALFF values of the left OFC were correlated with the color-word Stroop task performance (i.e. response errors) in the OGA group.

The ALFF maps of both the OGA group and control group are presented in Fig. 1, and the two groups both exhibited significantly higher ALFF values in the posterior cingulate cortex (PCC)/precuneus, medial prefrontal cortex (MPFC), and bilateral inferior parietal lobe (IPL) during the resting state. These regions are largely included in the default mode network in previous studies [19]. A two-sample *t*-test controlling for age and gender and corrected for multiple comparisons (using Monte Carlo simulations of the smallest cluster size yielding a corrected threshold of p<0.05 from an uncorrected threshold of p<0.005 for each voxel) revealed that the OGA group showed significant increases in ALFF values in the left medial OFC, left precuneus, left SMA, right PHG and bilateral MCC compared with the control group. No brain regions with decreased ALFF values were found. Additionally, a significantly positive correlation was observed between the duration of OGA and the standardized ALFF values in the left medial OFC (r  =  0.6627, *p*  =  0.0027) and left precuneus (r  =  0.5924, *p*  =  0.0096) (Fig. 2). The ALFF values of the left OFC were found to be correlated with the number of response errors during the incongruent condition among adolescents with OGA (r  =  0.6690, *p*  =  0.0024) (Fig. 3). Because the OGA subjects had significantly higher depression ratings measured by the BDI, we re-analyzed the functional imaging data, using the BDI as a covariate. The resulting data were similar to the original data. We also tested whether the BDI scores correlated with the ALFF values of the abnormal brain regions, duration of OGA and color-word Stroop task performance. However, no significant results were observed.

## Discussion

In the current study, the ALFF method was employed to investigate the resting state differences between patients with OGA and normal controls. ALFF is an easy and convincing method to measure the amplitude of the low-frequency fluctuations in the BOLD signal, and previous studies have shown the capacity of this method to precisely locate which brain region has abnormal spontaneous activity [13]. Within each group, we identified some regions exhibiting significantly higher ALFF values than other brain regions during the resting state (Fig. 1). These regions extensively overlapped with the major regions of the default mode network (DMN) [20]. With regards to the two sample *t*-test results, relative to the healthy controls, adolescents with OGA showed increased ALFF in the left medial OFC, left precuneus, left SMA, right PHG and bilateral MCC during the resting state (Fig. 2). It is worth noting that the OGA subjects had significantly higher depression ratings on the BDI, however, analysis including the BDI as a covariate revealed similar results. Furthermore, the ALFF values of left medial OFC and precuneus were positively correlated with the duration of OGA (Fig. 2). In addition, to validate the impaired cognitive control ability in adolescents with OGA, the color-word Stroop test was used in our study. Consistent with previous findings [14], [15], the OGA group committed more errors than the control group during the incongruent condition, which demonstrated that adolescents with OGA showed impaired cognitive control ability, as measured by the color-word Stroop test. Interestingly, the ALFF values of the left OFC were also correlated with the number of errors during the incongruent condition among adolescents with OGA (Fig. 3). Our results suggest that ALFF changes in the OFC may serve as a biomarker to reflect the impaired cognitive control ability of OGA.

In the present study, we found that ALFF values increased in the left medial OFC in the OGA group. Anatomically, the OFC has extensive connections with the striatum and limbic regions (such as the amygdala), which appear to be involved in cognitive control of goal-directed behavior through the assessment of the motivational significance of stimuli and the selection of behavior to obtain desired outcomes. The OFC’s structural abnormalities and dysfunction in OGA have been reported in previous studies [4], [11], [15]. Park et al. used a ^18^F-fluorodeoxyglucose positron emission tomography (PET) study to investigate regional cerebral glucose metabolism during the resting state in young individuals with OGA and normal controls, and showed that the OFC metabolic activity in adolescents with OGA was increased compared with normal controls [21]. This analysis suggested that the abnormal metabolic activity in the area of the OFC may be associated with impairment in impulse control and reward processing in adolescents with OGA. Regarding task-related functional MRI studies, Ko et al. identified the neural substrates of online gaming addiction via evaluation of the brain areas associated with the cue-induced gaming urge, and found that the OFC could be activated abnormally in addicts when compared with controls [22]. The similarity of this finding to the cue-induced craving in substance dependence [23], which suggested that the craving in gaming addiction and craving in substance dependence might share the same neurobiological mechanisms. Previous structural neuroimaging studies have also reported reduced gray matter volume of the OFC in the OGA group [1], [4]. In line with these functional and structural findings, our study found higher ALFF values in the medial OFC in adolescents with OGA compared with the controls. Moreover, a significant correlation between the ALFF values of the OFC and the task performance during the color-word Stroop test was observed in the OGA group (Figure 3). Previous addiction studies revealed association between Stroop interference and relative glucose metabolism in the OFC among cocaine-addicted subjects [24]. This brain-behavior relationship demonstrated that the abnormal resting state properties of the OFC were associated with impaired cognitive control ability among adolescents with OGA.

ALFF values were greater in the precuneus in OGA subjects compared to controls. The precuneus is a brain region in the posteromedial cortex of the parietal lobe and plays an important role in fundamental cognitive functioning [25]. The precuneus has been proposed to be involved in episodic memory retrieval, visual–spatial imagery, self-processing and consciousness [25]. Recently, some researchers also reported increased ReHo in the left precuneus in OGA college students compared with controls [11]. Moreover, a study showed that the precuneus was associated with a gaming urge, craving and severity of OGA, and suggested that the precuneus activates to process the gaming cue, integrate retrieved memory and contribute to cue-induced craving for online gaming [26]. Therefore, we suggest that the resting-state abnormalities of the precuneus in adolescents with OGA may be associated with craving in long-term OGA.

Greater ALFF values in OGA subjects, relative to controls, were also found in the left SMA, bilateral MCC and the right PHG. The SMA plays an important role in cognitive control, voluntary action, initiation/inhibition of motor responses [27] and also in emotional conflict [28]. The MCC is the middle part of the cingulate gyrus and critical for conflict monitoring and processing [29]. Previous substance use studies reported addiction-related resting state abnormalities of the SMA and MCC [30], [31]. The PHG is thought to contribute to the formation and maintenance of bound information in working memory [32]. Working memory refers to the temporary storage and on-line manipulation of information and is also crucial for cognitive control [33]. Liu et al. reported increased ReHo in the bilateral PHG in OGA college students compared with controls [11]. Moreover, some researchers also found lower fractional anisotropy of the PHG in OGA subjects [4]. Our results validated the abnormal resting state pattern of the PHG in the adolescents with OGA.

In conclusion, in the present study, we observed that ALFF was abnormal in adolescents with OGA compared to the controls, i.e. higher ALFF values in the left medial OFC, left precuneus, left SMA, right PHG and bilateral MCC. We also observed that the higher ALFF values in the left medial OFC and left precuneus were positively correlated with the duration of OGA. The ALFF values of the left OFC were correlated with the color-word Stroop task performance (i.e. response errors) in the OGA group. Our findings suggested that the abnormal spontaneous activity of these regions may reflect the underlying pathophysiology in OGA users. Due to the similar resting-state findings with drug addiction-related resting state changes, we suggested that OGA might share neural mechanisms with drug addiction. It is worth noting that depression should be considered as a potential confound when explaining the neuroimaging findings in the current study. A further comprehensive study is needed to provide more scientific perspectives about OGA.
